# Factors associated with kinesiophobia among myocardial infarction survivors: a biopsychosocial perspective

**DOI:** 10.3389/fpsyt.2025.1551758

**Published:** 2025-08-06

**Authors:** Xiaodan Zhang, Yamin Li, Qiuping Ma, Zheyi Jiang, Xinrui Han, Keming Yi, Lifang Cao

**Affiliations:** ^1^ Department of Cardiology, The Second Xiangya Hospital, Central South University, Changsha, China; ^2^ Clinical Nursing Teaching and Research Section, Second Xiangya Hospital, Central South University, Changsha, China

**Keywords:** biopsychosocial model, exercise rehabilitation, fear-avoidance model, kinesiophobia, myocardial infarction

## Abstract

**Objectives:**

This study aimed to examine the factors associated with kinesiophobia among myocardial infarction (MI) survivors.

**Methods:**

This study was based on the Biopsychosocial (BPS) model. A multicenter, descriptive, cross-sectional study was conducted at three tertiary hospitals in Changsha, Hunan Province, China, with MI survivors as participants. The Tampa Scale for Kinesiophobia Heart (TSK-SV Heart), Exercise Self-Efficacy Scale (ESES), Generalized Anxiety Disorder-7 (GAD-7), Patient Health Questionnaire-9 (PHQ-9), Self-Perceived Burden Scale (SPBS), the 10-Item Connor-Davidson Resilience Scale (CD-RISC-10), Family Resilience Assessment Scale (FRAS), Multidimensional Scale of Perceived Social Support (MSPSS) and Social Support Rating Scale (SSRS) were used to collect data. Hierarchical regression analysis was employed to identify significant predictors of kinesiophobia.

**Results:**

A total of 414 MI survivors participated. Hierarchical regression analysis showed that the total explanatory power of the model was 56.9%, with sociodemographic factors accounting for 17.0% and cognitive, behavioral, psychological and social support factors accounting for 39.9%. Kinesiophobia was significantly associated with age, gender, education level, disease duration, number of complications, exercise intensity, exercise duration, exercise frequency, exercise self-efficacy (ESE), depression, self-perceived burden (SPB), mental resilience, family resilience, perceived social support (PSS), and actual social support (ASS).

**Conclusions:**

This study identified several sociodemographic, psychosocial, and behavioral factors associated with kinesiophobia in MI survivors. Based on these findings, integrating psychological support, behavior-focused interventions, and strengthened social support systems may help reduce kinesiophobia in this population.

## 1 Introduction

Myocardial infarction (MI) is a significant public health problem that profoundly affects the well-being and quality of life of the population (1). Research indicates that the global prevalence of MI is 3.8% among individuals under 60 years old, rising to a staggering 9.5% among those over 60 years old (2). Exercise-based cardiac rehabilitation (CR), supported by scientific evidence, can effectively reduce cardiovascular mortality, recurrent cardiac events, and hospitalizations, while also enhancing health-related quality of life (3). Unfortunately, kinesiophobia is common among MI survivors and severely impacts their ability to engage in exercise rehabilitation (4).

Kinesiophobia, defined as an excessive and irrational fear of exercise or daily activities (5), has been identified as a considerable risk factor for physical-psychosocial dysfunctions among MI survivors (6). Individuals with high levels of kinesiophobia report reduced physical activity and shorter walking distances, as well as lower health-related quality of life (6). In addition, they tend to experience higher levels of dyspnea, comorbidities, anxiety, and depression (6). These negative outcomes may hinder long-term recovery and increase the risk of recurrent cardiac events (7). Hence, understanding the factors associated with kinesiophobia in MI survivors is crucial for nursing managers to make accurate assessments and implement appropriate interventions. However, existing research on kinesiophobia has primarily focused on patients with chronic pain and joint surgery (8, 9), with limited studies available on its impact in MI survivors. Additionally, age, education, self-efficacy, cardiac anxiety, depressive symptoms, and social support have been identified as independent risk factors for kinesiophobia (10–12). These findings, however, require validation in MI survivors, and existing studies generally lack a theoretical framework to guide them.

The Biopsychosocial (BPS) model recognizes that health and illness are the result of the dynamic interaction of biological, psychological, and social factors (13). These Psychosocial factors include cognitive, emotional, behavioral factors, and social support (13, 14). Following a MI, patients may develop an increased fear of physical activity due to catastrophic interpretations of pain or other bodily sensations. Negative emotional states and reduced self-efficacy can lead individuals to internalize this fear, resulting in maladaptive avoidance behaviors. Moreover, insufficient support from family, peers, or healthcare providers may further erode patients’ confidence in participating in physical activity. Collectively, these interrelated factors contribute to the avoidance of exercise and the development of kinesiophobia.

Self-efficacy is thought to play an important role in influencing individual behavior (15, 16). Exercise self-efficacy (ESE) represents an individual’s self-perception and evaluation of their exercise capabilities, serving as a significant predictor of exercise behavior (17). Research indicates that ESE is an independent predictor of kinesiophobia (18). Anxiety, depression, self-perceived burden (SPB), and resilience are widely recognized psychosocial variables. The relationship between anxiety, depression, and kinesiophobia seems clear, given that both anxiety and depression have been identified as independent factors influencing activity levels (19). SPB refers to the caregiving challenges resulting from an individual’s illness, encompassing concerns that can affect the caregiver and subsequently evoke negative emotions (20, 21). Studies suggest that elevated SPB strongly correlates with diminished rehabilitative exercise behavior, potentially leading individuals to adopt catastrophic perceptions of exercise (22). Resilience is conceptualized as the capacity to endure and recover from adversity (23, 24). As a positive potential, resilience independently influences psychological well-being; conversely, low resilience may provoke negative emotions, leading to an aversion to movement (23). Social support, as a socio-environmental factor, can be categorized into perceived social support (e.g., reassurance, encouragement) and actual social support (e.g., material assistance, informational guidance). It has been recognized as an independent determinant of physical activity (25). Hence, based on the above findings and BPS model, it is reasonable to assume that ESE, anxiety, depression, SPB, mental resilience, family resilience, perceived social support (PSS), and actual social support (ASS) are associated with kinesiophobia.

In summary, our study aimed to investigate the factors associated with kinesiophobia in MI survivors. We hypothesized that higher levels of anxiety, depression, and SPB would be associated with higher levels of kinesiophobia. In contrast, greater engagement in exercise behaviors, as well as higher levels of ESE, mental resilience, family resilience, PSS, and ASS would be associated with lower levels of kinesiophobia.

## 2 Materials and methods

### 2.1 Objectives

We aimed to examine the factors associated with kinesiophobia in MI survivors.

### 2.2 Study design

This study used a multicenter, descriptive, cross-sectional design.

### 2.3 Settings

The study was conducted at cardiovascular medicine outpatient clinics in three tertiary hospitals in Changsha, Hunan Province, China. These hospitals are affiliated with the same university (Central South University), and their annual number of cardiovascular medicine outpatient visits is similar.

### 2.4 Participants

The study participants consisted of survivors diagnosed with MI who sought treatment at the outpatient clinics of the Department of Cardiovascular Medicine in three tertiary hospitals affiliated with Central South University in Changsha, Hunan Province, China, between January and December 2023. Inclusion criteria were as follows: (1) Age ≥18 years; (2) Fulfillment of the diagnostic criteria for MI established by the European Society of Cardiology/American College of Cardiology Committee (26); (3) Cardiac function classification grade ≤ III; (4) Willingness to voluntarily participate in the study; (5) Ability to independently complete the questionnaire. Exclusion criteria included: (1) Patients with mobility disorders; (2) Patients with a history of significant physical, cognitive, or psychiatric disorders.

### 2.5 Ethical considerations

This study received approval from the Ethics Committee of the Second Xiangya Hospital of Central South University (2023118). Participants voluntarily agreed to take part in the study and were free to withdraw at any time. Before enrollment, all participants provided informed consent. Questionnaires were completed anonymously and collected immediately after completion. The allotted time for completing the questionnaire was 30 minutes, and all participants finished within the designated timeframe. As an incentive, each participant received a cell phone case and a keychain.

### 2.6 Data collection

Before starting this study, we received support from the Xiangya Medicine School of Central South University, as well as from the cardiovascular medicine outpatient departments of three hospitals. We fully informed all participants of the study’s purpose and assured them that it would be solely used for scientific research. Informed consent was obtained from all participants, and their personal information was anonymized to safeguard their privacy. To gauge the completion time for participants, we gathered 20 questionnaires as part of a pilot survey. Additionally, we refined the guidelines, informed consent form, and finalized the definitive version of the questionnaire.

### 2.7 Measures

Permission was obtained for all instruments used in this study. All measures used in this study showed good internal consistency. Participants were instructed to report their physical activity based on their experiences over the past month. Key variables in our study were identified based on the BPS model. The questionnaire comprised three sections: basic information (including sociodemographic characteristics, disease- and treatment-related factors, and exercise behavior), cognitive and psychological factors (including ESE, anxiety, depression, SPB, mental resilience, and family resilience), and social support factors (including PSS and ASS).

#### 2.7.1 Basic information of participants

The data collection sheets for basic information were self-designed. Specific details are provided in **Table 1**.

**Table 1:** Participants’ characteristics and univariate analysis of kinesiophobia (n = 414).

Variables	Category	n	%	Mean	SD	*t/F* statistic	*p*-value
Age				59.79	10.35	3.569	<0.001
	<60	205	49.5	45.19	5.15		
	≥60	209	50.5	43.44	4.80		
Gender	male	314	75.8	43.84	5.15	-3.676	0.001
	female	100	24.2	45.78	4.40		
Education level	less than high school	184	44.4	45.44	4.71	8.318	<0.001
	high school graduates	73	17.6	44.34	4.62		
	some college or vocational school	53	12.8	44.00	5.05		
	bachelor or higher	104	25.1	42.44	5.39		
Average monthly family income	<CNY ¥3000	35	8.5	45.57	4.62	6.579	0.002
	CNY ¥3000-5000	145	35.0	45.26	4.92		
	>CNY ¥5000	234	56.5	43.53	5.06		
Disease knowledge	very good	13	3.1	41.00	7.33	2.850	0.037
	good	225	54.3	44.06	4.83		
	fair	164	39.6	44.90	5.06		
	bad	12	2.9	44.50	4.78		
Number of implanted stents	0	229	55.3	44.01	5.06	1.543	0.189
	1	87	21.0	45.36	4.73		
	2	58	14.0	44.29	4.69		
	3	27	6.5	44.33	5.72		
	≥4	13	3.1	42.54	6.41		
Had a heart bypass surgery	Yes	13	3.1	44.69	9.98	-0.229	0.822
	No	401	96.9	44.33	4.83		
Disease duration (years)	<1	168	40.6	43.05	5.23	6.392	<0.001
	1-5	130	31.4	45.37	4.37		
	5-10	70	16.9	45.17	4.69		
	≥10	46	11.1	44.61	5.75		
Complication	Yes	356	86.0	44.54	4.99	2.369	0.018
	No	58	14.0	42.86	5.21		
Number of complications	0	58	14.0	42.86	5.21	6.569	<0.001
	1	119	28.7	43.18	5.24		
	2	124	30.0	44.45	4.72		
	3	82	19.8	45.94	4.54		
	≥4	31	7.5	46.45	4.77		
Combination of other cardiovascular diseases	Yes	54	13.0	45.28	5.00	-1.515	0.130
	No	360	87.0	44.16	5.04		
Hypertension	Yes	281	67.9	44.70	4.83	-2.331	0.020
	No	133	32.1	43.47	5.39		
Diabetes	Yes	157	37.9	44.54	4.96	-0.732	0.465
	No	257	62.1	44.17	5.10		
Exercise intensity	light intensity exercise	331	80.0	45.12	4.88	15.686	<0.001
	small intensity exercise	63	15.2	41.14	4.54		
	moderate intensity exercise	14	3.4	40.71	3.60		
	high intensity exercise	6	1.4	41.33	5.16		
Exercise duration	≤10 min	91	22.0	47.75	5.24	30.942	<0.001
	11–20 min	57	13.8	46.84	4.07		
	21–30 min	71	17.1	44.06	4.14		
	31–59 min	74	17.9	42.28	4.37		
	≥60 min	121	29.2	41.92	4.16		
Exercise frequency	≤ Once a month	34	8.2	45.85	5.51	11.113	<0.001
	2–3 times per month	42	10.1	46.67	4.86		
	1–2 times per week	57	13.8	45.25	5.18		
	3–5 times per week	69	16.7	45.96	4.78		
	once per day	212	51.2	42.81	4.60		

#### 2.7.2 Kinesiophobia

The Tampa Scale for Kinesiophobia Heart (TSK-SV Heart) was adapted by Dr. Bäck in 2012 from the TSK designed for pain patients (27). We used the Chinese version of the TSK-SV Heart to assess kinesiophobia in patients in our study (28). The scale was scored using a 4-point Likert scale, where 1 represents ‘strongly disagree’ and 4 represents ‘strongly agree’. The total score ranges from 17 to 68, with higher scores indicating higher levels of exercise fear. The TSK-SV Heart has been reported to exhibit good internal consistency and reliability in patients with coronary artery disease (4).

#### 2.7.3 ESE

We used the Exercise Self-Efficacy Scale (ESES) to assess individuals’ subjective confidence in successfully completing challenging exercises, as adapted into Chinese by Tung et al. (29). This scale comprises 18 items. The total score ranges from 0 to 100, with scores below 50 indicating low exercise self-efficacy (ESE) and higher scores indicating greater ESE. The ESES reported to exhibit good psychometric properties in patients after percutaneous coronary interventions (30).

#### 2.7.4 Anxiety

The Chinese version of the Generalized Anxiety Disorder-7 (GAD-7) was used as a screening tool to assess patients’ generalized anxiety and symptom severity (31). This user-friendly scale consists of 7 items, each assigned a score from 0 to 3, resulting in a total score range of 0 to 21. Higher scores indicate higher levels of anxiety and more severe symptoms. The GAD-7 scale has been reported to exhibit good psychometric properties in populations with coronary heart disease (32).

#### 2.7.5 Depression

The Chinese version of the Patient Health Questionnaire (PHQ-9) is a simple and effective tool for screening depression, now widely used in clinical practice (33, 34). It consists of 9 items, each assigned a score from 0 to 3, resulting in a total score range of 0 to 27. Higher scores indicate a higher likelihood of depression. The PHQ-9 has been shown to possess acceptable psychometric properties for screening and identifying current depressive episodes in patients with coronary artery disease (35).

#### 2.7.6 SPB

The Chinese version of the Self-Perceived Burden Scale (SPBS) was used in this study to assess the SPB experienced by patients (36). This scale consists of 10 items, each rated on a scale from 1 to 5. Higher scores indicate a higher self-perceived burden. The SPBS has been reported to exhibit good psychometric properties in patients with coronary heart disease following percutaneous coronary intervention (37).

#### 2.7.7 Mental resilience

The Chinese version of the 10-Item Connor-Davidson Resilience Scale (CD-RISC-10) was used to assess patients’ mental resilience (38). Items are rated on a 5-point scale from 0 (not at all true) to 4 (almost always true), yielding a total score range from 0 to 40. Higher scores indicate higher levels of mental resilience. The CD-RISC-10 has been shown to possess good psychometric properties in adolescent and young adult heart transplant recipients (39).

#### 2.7.8 Family resilience

Family resilience in MI survivors was assessed using the Chinese version of the Family Resilience Assessment Scale (FRAS) developed by Pu et al. (40). This scale consists of 20 items, categorized into four dimensions: perseverance, tolerance, openness, and competence. The score range for this scale is 20 to 100, with higher scores indicating greater levels of family resilience. The FRAS has demonstrated good psychometric properties for assessing family resilience in the context of adversity (41).

#### 2.7.9 PSS

The Chinese version of the Multidimensional Scale of Perceived Social Support (MSPSS) was used in this study (42). The scale consisted of 12 items, primarily assessing the social support perceived and received by participants in three dimensions: family support, friend support, and other support. The scale uses a 7-point Likert scale, with each item scored from 1 to 7, ranging from ‘strongly disagree’ to ‘strongly agree’. Higher scores indicate higher levels of perceived social support (PSS). The MSPSS has been shown to possess good psychometric properties in populations with coronary heart disease (43).

#### 2.7.10 ASS

The Chinese version of the Social Support Rating Scale (SSRS) was used to assess the level of ASS among MI survivors (44). This scale consists of 10 items and includes three dimensions: objective support (items 1, 3, 4, and 5), subjective support (items 2, 6, and 7), and the utilization of social support (items 8 to 10). The total score on the scale ranges from 12 to 66, with higher scores indicating higher levels of ASS. The SSRS has been shown to possess good psychometric properties in populations with coronary heart disease (45).

### 2.8 Sample size

In total, we included 414 study participants. Ideally, a linear regression analysis requires a recommended number of subjects ranging from 5 to 20 per variable, or a sample size of at least 200 subjects to meet the requirements of any regression analysis (46). In our analysis, the multiple linear regression model included 25 variables, each with a sample size of more than 16 subjects. Thus, the number of subjects was considered adequate. Additionally, our study did not have any missing data that needed to be addressed.

### 2.9 Statistical analysis

Data analysis was conducted using SPSS statistical software version 25.0 (IBM Corp., Armonk, NY, USA). A two-tailed *p*-value of less than 0.05 was considered statistically significant, and all tests were two-tailed. Descriptive statistics were used to determine the distribution of sociodemographic characteristics, disease- and treatment-related factors, exercise behavior, and key study variables of the participants. The mean and standard deviation (SD) were used to report continuous variables, while categorical variables were presented as frequencies and percentages.

Differences in kinesiophobia based on categorical variables were analyzed using independent t-tests and one-way ANOVA with *post-hoc* Scheffé’s test. The Pearson correlation coefficient was used to examine relationships among study variables. Subsequently, a hierarchical multiple regression analysis was conducted to identify the multidimensional factors influencing kinesiophobia in MI survivors. All variables significantly associated with the outcome variable were included in the respective hierarchical regression analysis. Based on the results of prior bivariate analyses, four-step models were used, encompassing participant characteristics, cognitive and behavioral factors (including exercise intensity, exercise duration, exercise frequency, and ESE), psychological factors (including anxiety, depression, SPB, mental resilience, and family resilience), and social support factors (including PSS and ASS). Statistical significance was set at a *p*-value of less than 0.05.

## 3 Results

### 3.1 Participant characteristics

**Table 1** presents the participant characteristics. The mean age of all participants was 59.79 years (SD: 10.35), ranging from 31 to 88 years. The majority of participants were male (N = 314; 75.8%). Approximately 25.1% of individuals had attained a bachelor’s degree or higher. Over half of the households had an average monthly income exceeding CNY ¥5000 (N = 234; 56.5%). Notably, more than 57% of participants considered themselves well-informed about the disease. Slightly over 50% of participants did not have a stent implanted (N = 229; 55.3%). The vast majority had not undergone bypass surgery (N = 401; 96.9%). Hypertension was present in 67.9% of participants, and more than one-third had diabetes mellitus (N = 157; 37.9%). Roughly 80% engaged in light-intensity exercise, with over 70% exercising for less than 60 minutes per session. More than half of the patients exercised once per day (N = 212; 51.2%).

### 3.2 Descriptive statistics of kinesiophobia and related variables

**Table 2** presents the means and SDs of the 9 measures in the current sample.

**Table 2:** Descriptive statistics of kinesiophobia and related variables in MI survivors.

Scales	Score range	Mean	SD	Cronbach’s α
TSK-SV Heart-C	17-68	44.31	5.05	0.878
ESES	0-100	46.77	7.49	0.961
GAD-7	0-21	10.42	3.67	0.929
PHQ-9	0-27	12.51	3.42	0.814
SPBS	10-50	20.21	6.85	0.950
CD-RISC-10	0-40	34.12	8.05	0.945
FRAS	20-100	74.68	18.21	0.981
MSPSS	12-84	55.08	16.50	0.968
SSRS	12-66	40.02	11.17	0.894

SD, standard deviation.

### 3.3 Differences in participants characteristics in kinesiophobia

The level of kinesiophobia showed significant associations with age, gender, education level, average monthly family income, disease knowledge, disease duration, complications (yes/no), number of complications, hypertension (yes/no), exercise intensity, exercise duration, and exercise frequency (all *p* < 0.05) (**Table 1**).

### 3.4 Relationship between kinesiophobia and related variables

**Table 3** presents a summary of the correlations between kinesiophobia and related variables among MI survivors. Kinesiophobia demonstrated a positive correlation with anxiety (*r* = 0.340; *p* < 0.001), depression (*r* = 0.468; *p* < 0.001), SPB (*r* = 0.430; *p* < 0.001), and a negative correlation with ESE (*r* = -0.534; *p* < 0.001), mental resilience (*r* = -0.460; *p* < 0.001), family resilience (*r* = -0.519; *p* < 0.001), PSS (*r* = -0.580; *p* < 0.001), and ASS (r = -0.545; *p* < 0.001).

**Table 3:** Correlation between kinesiophobia and related variables in MI survivors.

Variables	1	2	3	4	5	6	7	8	9
1. Kinesiophobia	1								
2. ESE	-0.534**	1							
3. Anxiety	0.340**	-0.337**	1						
4. Depression	0.468**	-0.384**	0.626**	1					
5. SPB	0.430**	-0.364**	0.374**	0.435**	1				
6. Mental resilience	-0.460**	0.217**	-0.118**	-0.170**	-0.164**	1			
7. Family resilience	-0.519**	0.290**	-0.212**	-0.293**	-0.239**	0.535**	1		
8. PSS	-0.580**	0.341**	-0.163**	-0.259**	-0.268**	0.641**	0.651**	1	
9. ASS	-0.545**	0.315**	-0.192**	-0.282**	-0.248**	0.519**	0.623**	0.740**	1

**P <0.01. ESE, exercise self-efficacy; SPB, self-perceived burden; PSS, perceived social support; ASS, actual social support.

### 3.5 Factors associated with kinesiophobia in MI survivors

Hierarchical regression analysis was performed to identify factors associated with kinesiophobia in MI survivors. Statistically significant variables identified in the univariate analysis were included in the regression models. Model 1 incorporated sociodemographic characteristics of participants as independent variables, accounting for 17.0% of the variance in kinesiophobia among MI survivors. In Model 2, cognitive and behavioral factors (including exercise intensity, exercise duration, exercise frequency, and ESE) were introduced at the second level, resulting in a 22.5% increase in explanatory power. Model 3 included psychological factors (including anxiety, depression, SPB, mental resilience, and family resilience) at the third level, leading to a significant 14.9% enhancement in the explanatory power of the overall model. Furthermore, the inclusion of social support factors (including PSS and ASS) in Model 4 increased the explanatory power of the overall regression model by only 2.5%. The comprehensive model demonstrated a total explanatory power of 56.9%. Age, gender, education level, disease duration, number of complications, exercise intensity, exercise duration, exercise frequency, ESE, depression, SPB, mental resilience, family resilience, PSS, and ASS were significant influencing factors of kinesiophobia among MI survivors (**Table 4**).

**Table 4:** Hierarchical multiple linear regression analysis of factors associated with kinesiophobia in MI survivors.

Model	B	SE	*β*	*t*	*P*	95% *CI*		R	R^2^	R^2^(adj)
						Low Up				
**Model 1**								0.434	0.188	0.170
Constants	44.387	2.107		21.068	<0.001	40.245	48.529			
Age	-2.346	0.465	-0.233	-5.049	<0.001	-3.260	-1.433			
Gender	1.411	0.551	0.120	2.561	0.011	0.328	2.495			
Education level	-0.815	0.237	-0.201	-3.438	<0.001	-1.281	-0.349			
Disease duration (years)	0.724	0.232	0.145	3.115	0.002	0.267	1.181			
Number of complications	1.092	0.278	0.247	3.933	<0.001	0.546	1.638			
**Model 2**								0.644	0.414	0.395
Constants	49.760	1.961		25.372	<0.001	45.905	53.616			
Exercise intensity	-0.811	0.365	-0.095	-2.218	0.027	-1.529	-0.092			
Exercise duration (min)	-0.636	0.204	-0.193	-3.118	0.002	-1.037	-0.235			
Exercise frequency	0.540	0.206	0.143	2.619	0.009	0.135	0.946			
Exercise self-efficacy	-0.070	0.010	-0.411	-7.011	<0.001	-0.090	-0.051			
**Model 3**								0.751	0.564	0.544
Constants	52.084	2.096		24.847	<0.001	47.963	56.205			
PHQ-9	0.214	0.070	0.145	3.071	0.002	0.077	0.351			
SPB	0.085	0.031	0.115	2.717	0.007	0.023	0.146			
Mental resilience	-0.118	0.025	-0.188	-4.648	<0.001	-0.168	-0.068			
Family resilience	-0.057	0.012	-0.206	-4.945	<0.001	-0.080	-0.034			
**Model 4**								0.768	0.590	0.569
Constants	52.385	2.060		25.428	<0.001	48.334	56.435			
PSS	-0.050	0.018	-0.163	-2.852	0.005	-0.084	-0.016			
ASS	-0.051	0.023	-0.112	-2.217	0.027	-0.096	-0.006			

Model1: F=10.415, P<0.001;Model 2: F=21.774, P<0.001;Model 3: F=28.363, P<0.001; Model 4: F=28.308, P<0.001.

Model 1 incorporated sociodemographic characteristics of participants as independent variables. Model 2 incorporated cognitive and behavioral factors (including exercise intensity, exercise duration, exercise frequency, and exercise self-efficacy) of participants as independent variables. Model 3 incorporated psychological factors (including anxiety, depression, SPB, mental resilience, and family resilience) of participants as independent variables. Model 4 incorporated social support factors (including PSS and ASS) of participants as independent variables.

## 4 Discussion

This study employed a cross-sectional, multicenter design to examine the factors associated with kinesiophobia among MI survivors. This is the first study in China to explore the factors influencing kinesiophobia in MI survivors based on the frameworks of BPS model. This study provides valuable data to elucidate the factors influencing kinesiophobia in MI survivors and offers new perspectives for public health administration to develop effective intervention strategies for kinesiophobia.

### 4.1 Relationships between kinesiophobia and sociodemographic characteristics

First, our investigation revealed a relationship between age and kinesiophobia levels. Specifically, kinesiophobia levels decreased with increasing age. This finding is inconsistent with the results of a study by Cai et al. (2018), which indicated that older age is associated with heightened kinesiophobia, particularly in individuals aged 76 and above (10). However, another study investigating Polish patients with coronary artery disease noted that age had no relationship with physical activity and kinesiophobia (11). The differences in the results of these studies may be related to cultural background, sample size, and age ranges. Therefore, future longitudinal studies with larger sample sizes are needed to validate the causal relationship between age and kinesiophobia.

Second, we found that female MI survivors exhibited higher levels of kinesiophobia compared to their male counterparts. This finding is consistent with previous research demonstrating sex-based differences in pain perception and emotional responses to pain (47, 48). For instance, Presto et al. (2022) reported that women show heightened pain sensitivity across multiple levels of the neuraxis, indicating potential biological mechanisms underlying stronger fear-related responses (47). In addition, Racine et al. (2012) conducted a systematic review of a decade of experimental pain research and concluded that women tend to report higher pain intensity and greater affective responses than men, further supporting the presence of meaningful sex differences in pain experiences (48). Together, these findings reinforce our observation that female MI survivors may be more susceptible to elevated kinesiophobia following cardiac events.

Third, our findings indicated that MI survivors with lower levels of education exhibited higher levels of kinesiophobia. This result aligns with the findings reported by Knapik et al. (11). Educational attainment has been identified as one of the most influential determinants of health literacy (49). Additionally, health literacy plays a critical role in shaping various health-related outcomes, including overall health status, quality of life, clinical indicators, health behaviors, and engagement with preventive services (49). Individuals with limited health literacy tend to report greater pain severity and elevated levels of kinesiophobia (50). These findings suggest that promoting health literacy across the population, as well as improving the accessibility and comprehensibility of health services, may serve as effective strategies to reduce kinesiophobia.

Furthermore, we found that disease duration independently influences kinesiophobia. When the disease duration reached one year or more, kinesiophobia levels decreased as the duration increased. This phenomenon may be attributed to patients overcoming their fears related to acute cardiac events and experiencing the benefits of consistent exercise. In addition, we found that the number of complications affects kinesiophobia levels. Specifically, the higher the number of complications, the higher the level of kinesiophobia. Studies have shown that increased complications in patients with MI can lead not only to difficulties in emotional adjustment, but also to physical consequences, such as activity limitations and increased pain (51–53). These physical factors, especially elevated pain levels and decreased fitness, may further reinforce avoidance behaviors and lead to higher levels of exercise fear (54). In addition, the severity of MI may be associated with a higher number of comorbidities, which may exacerbate the patient’s physical and psychological burden, limiting their ability and confidence to engage in physical activities (6, 55).

### 4.2 Relationships between kinesiophobia and cognitive and behavioral factors

Our results indicated that exercise intensity, duration, and frequency independently influence kinesiophobia. Specifically, increased exercise intensity and duration were found to correlate with reduced kinesiophobia. Additionally, the data suggested that MI survivors who engage in daily exercise exhibit the lowest levels of kinesiophobia. This finding indicates that higher levels of physical activity are associated with lower levels of kinesiophobia. Therefore, improving exercise adherence and encouraging regular physical activity in patients may be an effective strategy to reduce kinesiophobia and improve rehabilitation outcomes.

In our study, we found that ESE was a significant predictor of kinesiophobia. Specifically, lower levels of ESE were associated with higher levels of kinesiophobia. This finding is consistent with the research conducted by Marques-Sule et al. among heart transplant recipients (18). It is possible that a bidirectional relationship exists between ESE and kinesiophobia, as both low self-efficacy and fear of movement are recognized barriers to physical activity (56). Reduced ESE may weaken an individual’s motivation and confidence to engage in physical activity, thereby contributing to increased levels of kinesiophobia. Conversely, heightened kinesiophobia may lead to avoidance of exercise, which in turn further erodes one’s belief in their ability to be physically active.

### 4.3 Relationships between kinesiophobia and psychosocial factors

The results of this study suggested that depression was significantly associated with kinesiophobia among MI survivors. This finding aligns with the results reported by Ebina et al. in patients experiencing pregnancy-related lumbopelvic pain during late pregnancy (57). Prior research has identified a bidirectional association between depression and physical activity (58). Individuals experiencing depression may reduce their physical activity, which in turn increase fear of pain and foster catastrophic thinking, potentially contributing to the development of kinesiophobia (59, 60). Conversely, kinesiophobia may also exacerbate depressive symptoms by limiting physical activity and reinforcing a sedentary lifestyle (61). These associations highlight the importance of recognizing and addressing depressive symptoms when managing kinesiophobia in MI survivors.

Our study demonstrated that SPB, as a predictor of kinesiophobia, was positively associated with kinesiophobia. We previously disclosed that depression was a predictor of higher levels of kinesiophobia among MI survivors. Undoubtedly, high levels of depression lead to higher SPB in individuals (62). This finding may suggest an association between depression, SPB, and kinesiophobia. Future longitudinal studies are needed to explore these potential causal pathways more thoroughly. Additionally, it has been proposed that individuals with a high perception of self-burden should improve their self-management behaviors, particularly in relation to rehabilitative exercise management (22).

Our findings indicated that mental resilience and family resilience were significantly associated with kinesiophobia. Although there is a lack of studies directly investigating the role of resilience in kinesiophobia among MI survivors, prior research has found that lower levels of resilience are correlated with increased pain and functional impairment in populations undergoing hip preservation (63). Moreover, both mental and family resilience have been recognized as protective factors for mental health (23, 64). Given the well-established relationship between mental health and physical activity (65, 66), it is plausible that low resilience may be linked to heightened emotional distress and reduced engagement in physical activity, which may contribute to greater fear of movement. While these associations do not establish causality, they highlight the potential relevance of psychological resilience in understanding and managing kinesiophobia.

### 4.4 Relationships between kinesiophobia and social support factors

The results of this study suggested that social support was an influencing factor of kinesiophobia among MI survivors. When both PSS and ASS were included in the hierarchical regression model, they jointly explained 2.5% of the variance in kinesiophobia. According to the Fear-Avoidance Model (FAM), low levels of social support may contribute to the development of excessive fear of movement in some individuals (7). In our study, we observed that lower levels of both PSS and ASS were correlated with higher levels of kinesiophobia. Therefore, we recommend that public health policymakers and nursing managers develop targeted support strategies to address the specific needs of MI survivors experiencing elevated levels of kinesiophobia. These strategies could include professional rehabilitation tutorials (delivered online or in person), peer support groups, psychological counseling services, and the integration of community healthcare resources (67).

## 5 Conclusions

This study, based on the frameworks of the BPS model, identified multiple factors that predict kinesiophobia in MI survivors. These factors were determined through stratified regression analysis and include age, gender, education level, disease duration, number of complications, exercise intensity, exercise duration, exercise frequency, ESE, depression, SPB, mental resilience, family resilience, PSS, and ASS. Despite these findings, theoretical and conceptual frameworks are underutilized in designing interventions for MI survivors with kinesiophobia. The factors identified in this study can help guide nursing managers in developing interventions to improve exercise rehabilitation and reduce kinesiophobic behaviors. Notably, interventions that focus on enhancing mental health, family resilience, and social support for MI survivors are likely to lead to favorable outcomes.

## 6 Implications

First, we identified several sociodemographic and behavioral factors that influence kinesiophobia in MI survivors, including age, gender, education level, disease duration, number of complications, exercise intensity, exercise duration, and exercise frequency. These findings highlight the importance of proactive assessment in clinical settings. Nurse managers, in particular, can play a key role in evaluating these factors to better identify patients who may be at risk of experiencing difficulties with participating in exercise-based rehabilitation programs. Second, factors such as ESE, depression, SPB, mental resilience, family resilience, PSS, and ASS significantly impact kinesiophobia in MI survivors. These findings underscore the importance of positive psychological interventions, behavioral therapy, and social support in achieving favorable clinical outcomes for MI survivors. Lastly, the public sector plays a crucial role in alleviating kinesiophobia among MI survivors, and its management and intervention require a collaborative, multidisciplinary approach. The strengths of the public sector should be leveraged to enhance patient care, optimize resource allocation, and foster effective teamwork.

## 7 Limitations

This study has some limitations. First, the utilization of a cross-sectional study design restricts the ability to establish causal relationships between variables. Hence, future research should consider employing a longitudinal design to examine the trajectory of kinesiophobia among MI survivors over time, as well as changes in its associated factors. Second, we did not collect data regarding patients’ pain levels. Given the established bidirectional relationship between pain and kinesiophobia, future research should include pain assessment to better clarify its potential role in this relationship. Third, there may exist additional factors that influence kinesiophobia in MI survivors, such as personality traits, pain perception, symptoms at disease onset, type of MI, and cardiac functional classification. This may lead to a limited explanation of the factors influencing kinesiophobia among MI survivors in this study.

Finally, we did not assess variations in patients’ post-MI activity restrictions or the education they received regarding safe levels and timing of exercise. These factors may influence kinesiophobia and should be explored in future research.

## Data Availability

The raw data supporting the conclusions of this article will be made available by the authors, without undue reservation.
